# Potential carcinogenic role of Reg IV in ulcerative colitis-associated colorectal neoplasia

**DOI:** 10.3332/ecancer.2024.1751

**Published:** 2024-08-29

**Authors:** Yosra Abdelmonem Zamzam, Yomna Zamzam, Ayman Elsaka, Lamiaa Al Fadaly, Tamer Haydara, Alaa Ibraheem Amer

**Affiliations:** 1 Department of Clinical Pathology, Faculty of Medicine, Tanta University, Tanta 31111, Egypt; 2 Department of Pathology, Faculty of Medicine, Tanta University, Tanta 31111, Egypt; 3 Clinical Pathology, National Cancer Institute, Cairo University, Giza 12511, Egypt; 4 Department of Internal Medicine, Faculty of Medicine, Kafr El Sheikh University, Kafr El-Sheikh 33511, Egypt; a https://orcid.org/0000-0003-0270-3140

**Keywords:** ulcerative colitis, neoplasia, dysplasia, colorectal cancer, Reg IV, PCR

## Abstract

**Background:**

Early detection of ulcerative colitis-associated neoplasia (UC-N) remains a clinical challenge. Identification of molecular biomarkers for colorectal dysplasia and cancer may be extremely beneficial in early detection and managing cancer risk in long-standing ulcerative colitis (UC) patients.

**Objective:**

The aim of this work is to investigate the role of Reg IV in comparison to P53 and KRAS in UC-associated dysplasia and colorectal cancer (CRC) in order to evaluate the potential use of Reg IV for dysplasia and cancer screening in UC patients.

**Methods:**

The study was conducted on 5 groups each 20 colonic endoscopic samples: 1) Normal colonic mucosa, 2) Active UC without dysplasia/carcinoma, 3) UC-associated dysplasia, 4) UC-associated CRC (UC-CRC), 5) Sporadic CRC. All included cases were subjected to Reg IV mRNA expression analysis by quantitative reverse transcription polymerase chain reaction, and immunostaining for Reg IV, P53 and KRAS.

**Results:**

Reg IV mRNA expression levels were found to be significantly higher in groups 3 and 4 (mean: 3.37 and 5.70, respectively). Reg IV immunostaining was highly expressed in groups 3 and 4 (mean: 45.80 and 62.35, respectively). While P53 and KRAS immunostaining was highly expressed in group 5 (mean: 64.57 and 62.90). Furthermore, Reg IV immunoexpression had shown a negative correlation with P53 and KRAS immunoexpression in groups 4 and 5.

**Conclusion:**

Higher expression of Reg IV in patients with UC-dysplasia and UC-CRC versus KRAS and P53 expression in sporadic CRC, suggests a potential role of Reg IV in UC carcinogenesis pathway. This could advocate the use of Reg IV as a screening biomarker for UC-N among patients with long-standing UC as well as a promising targeted therapeutic strategy.

## Introduction

Ulcerative colitis (UC) is defined as a chronic relapsing inflammatory bowel disease that affects the colon and rectum, leading to continuous mucosal inflammation [1]. The incidence of UC-associated neoplasia (UC-N) including both dysplasia and cancer increases in patients who have long-standing and extensive UC. In order to improve the prognosis of UC-associated colorectal cancer (UC-CRC), it is significant to diagnose it at an early or precancerous state in patients with long-duration UC [2]. 

Although UC-CRC accounts for just 1%–2% of colorectal cancers (CRCs) in the general population, it contributes to nearly 15% of all deaths in UC patients. Thus, it is critical to identify high-risk patients and carry out the appropriate monitoring [3].

The pathogenesis of UC-CRC is considered a multifactorial process, starting from no dysplasia to irreversible dysplasia, followed by UC-associated dysplasia (UC-dysplasia) which is divided into low-grade and high-grade dysplasia leading eventually to carcinoma [4]. Various immunological and genetic alterations, including oncogene overexpression, tumor suppressor gene inactivation and mutations, are involved in the oncogenesis process [5].

A specific series of changes in tumor suppressor genes and oncogenes that are commonly observed in sporadic CRC (S-CRC) may be also significant in the carcinogenesis of UC-CRC [6]. TP53, a tumor suppressor gene and KRAS, a proto-oncogene, have been strongly implicated in S-CRC [7]. Nevertheless, involved gene sequences and mutation frequencies of TP53 and KRAS can be different between UC-CRC and S-CRC, their sequence of events may also differ. Instead of the typical adenoma-carcinoma sequence, UC-CRC develops with an inflammatory-dysplasia-carcinoma sequence [8].

Regenerating islet-derived family, member 4 (Reg IV) is a member of the Reg gene family, belonging to the calcium-dependent lectin superfamily [9]. Reg IV is a secretory protein that is expressed in various normal tissues, including the pancreas, small intestine, colon and stomach [10]. Increased expression of Reg IV has been found in inflammatory bowel diseases [11] and various cancerous tissues such as CRC [12], gastric cancer [13], prostate cancer [14] and pancreatic cancer [15].

For the early detection of dysplasia, it is crucial that the etiology of CRC development in UC patients can be understood. Moreover, understanding the differences in carcinogenesis between UC-CRC and S-CRC could offer a new tool for targeted therapy in patients with UC-CRC [16].

Colonoscopy is mainly recommended for surveillance; however, because of the inflammatory and regenerative changes in the colonic mucosa, endoscopic and histologic diagnosis of dysplasia is often challenging [17]. Consequently, an objective molecular biomarker for dysplasia and cancer screening would be beneficial for cancer risk management in UC patients.

In this study, we sought to investigate the role of Reg IV in comparison to P53 and KRAS in UC-associated dysplasia and CRC in order to evaluate the potential use of Reg IV for dysplasia and cancer screening in patients with long-standing UC.

## Material and methods

### Case selection

This prospective study was conducted from May 2021 to 2023, among Internal medicine, Pathology and Clinical Pathology Departments, Kafr El Sheikh University and Tanta University, Egypt.

During this period, endoscopic samples were selected from patients with long-standing UC more than 10 years either for follow-up or complaining of rectal bleeding, chronic diarrhea and abdominal pain. Besides, endoscopic samples from patients presented with clinically suspicious colonic masses without a history of UC or hereditary CRC were also included for S-CRC sample selection.

### Sample collection

Two separate endoscopic samples were obtained from the same lesion in all included patients. One sample was for histopathological examination and the other for molecular study. After histopathological examination of colonoscopic tissue samples, the cases were divided into five groups each with 20 samples:

1. Normal colonic mucosal specimens (control) were obtained from the colonic mucosa which showed no evidence of histological inflammation.
2. Active UC without dysplasia/carcinoma group showed evidence of neutrophils in the lamina propria, crypt abscess and crypt distortion.
3. UC-dysplasia group. Dysplasia was defined as any of altered nuclear/cytoplasmic ratio, increased cell size and/or an increase in mitotic figures.
4. UC-CRC group 5) S-CRC group.

Groups 1 and 2 were considered non neoplastic cases and the other groups were neoplastic cases. The clinicopathological features such as age at time of operation, sex of the patients and site of the lesion, were collected from the medical records.

All included cases were subjected to quantitative reverse transcription polymerase chain reaction (qRT‐PCR) for Reg IV mRNA expression analysis and immunostaining for Reg IV, P53 and KRAS.

### Inclusion criteria

1) Patients with long-standing UC more than 10 years for endoscopic surveillance, 2) UC patients complaining of rectal bleeding, chronic diarrhea or abdominal pain, 3) UC patients with clinically suspicious colonic mass, 4) individuals undergoing a colonoscopy to check for CRC and 5) patients ranging in age from 35 to 75 years old.

### Exclusion criteria

Patients with any of the following were excluded from the study; 1) other type of colitis other than UC, 2) hereditary CRC including familial adenomatous polyposis or hereditary nonpolyposis CRC, 3) family history of CRC 4) use of nonsteroidal anti-inflammatory medicines in the last 3 months and 5) history of other malignant tumors, autoimmune disease or other chronic inflammatory disease.

### Ethics approval and consent to participate

This study was approved by the ethics committee of the Faculty of Medicine, Tanta University (approval code, 36264PR492) in accordance with the Declaration of Helsinki for experiments in humans and written informed consent was obtained from each participant.

### Histopathological evaluation

After colonoscopy, tissue samples obtained for histopathological examination were fixed in formalin, embedded as paraffin blocks and sectioned at 4 μm, and stained with eosin and hematoxylin.

### Immunohistochemistry procedure

All slides were briefly rehydrated and antigen retrieval was carried out by sodium citrate (pH = 6.0) in a pressure cooker (EDTA buffer, pH = 8.4). Endogenous peroxidase combined with 3% hydrogen peroxide was used to block all slides, whereas 2.5% bovine serum albumin in phosphate-buffered saline was used to block non-specific protein.

The following primary antibodies were utilized: anti-REG IV (1:50; R&D Systems, Minneapolis, MN, USA), anti-P53 (1:50; clone DO-7, cat # M7001, Dako) and anti-KRAS mouse monoclonal (1:10; Clone-F234, Santacruz biotechnology) and counterstained with hematoxylin.

The degree of immunostaining of formalin-fixed, paraffin-embedded sections was scored and reviewed independently by three different pathologists. Cytoplasmic with or without nuclear localization for Reg IV, cytoplasmic localization KRAS and nuclear localization for P53 are considered positive. Staining was categorized as negative, score 0 (0-<10%) or positive and scored as the overall proportion of cells (score 1: 10%–50% and score 2: >50%]. Quantitative calculation in the area of maximum staining per 10 high power fields using image analysis software (image J software).

### Quantitative reverse transcription polymerase chain reaction

Extraction of total RNA from frozen tissue samples by utilizing RNA extraction kits (RNeasy FFPE Kit, Qiagen, Germany) strictly following the instructions of manufacture. The concentrations of RNA were quantitatively measured at 260 nm absorbance using NanoDrop 2000/2000c Spectrophotometer (Thermo Scientific, USA). QuantiTect^®^ Reverse Transcription (Qiagen, Germany) was conducted to reverse transcribe the isolated RNA to cDNA. Reg IV mRNA expression was relatively quantified using TaqMan assay kits (Thermo Scientific, USA).

The primers used in the study: for Reg IV: forward:5′-CAGATCCTGGTCTGGCAAGT-3′ and reverse: 5′- ATTCGTTGCTGCTCCAAGTT-3′. Internal control GAPDH was used for each sample (forward primer: 5′-ACCACAGTCCATGCCATCCAC-3′; reverse primer: 5′-TCCACCACCCTGTTGCTGTA-3′). The plate was applied on real-time PCR system (Applied Biosystems, Canada) including the following thermal profile: holding at 95°C for 10 seconds then 45 cycles of denaturation at 95°C for 15 seconds, annealing at 60°C for 30 seconds and extension at 72°C for 30 seconds. The cycle threshold (CT) was gained for the gene using Applied Biosystems, step I version, software analysis modules, Relative mRNA expression levels were determined using the 2^−ΔΔCt^ method [18].

### Statistical analysis

Data were fed to the computer and analyzed by IBM SPSS software package version 20.0. (Armonk, NY: IBM Corp). Categorical data were represented as percentages and numbers. A chi-square statistical test was used to compare categorical proportions. Quantitative data were expressed as mean, standard deviation, range (minimum and maximum) and median. One-way ANOVA test was used to compare the different studied groups. Kruskal Wallis test was used to compare different groups for not normally distributed quantitative variables. Correlation between two normally distributed quantitative variables was conducted by the Pearson coefficient. The receiver operating characteristic curve (ROC) was utilized to assess the sensitivity and specificity of quantitative diagnostic measures between different groups. The area under the curve (AUC) is the area between the curve and the reference line, it represents how the markers are capable of distinguishing between the studied groups.

## Results

The clinico-pathological characteristics of the five studied groups were shown in Table 1. The predominant sex in all studied groups was males (50%–80%). Regarding location, the rectum and left colon were the most common sites for UC and UC-dysplasia groups (40% and 35%, respectively), the right colon for UC-CRC [45%] and the left colon for S-CRC (50%). The mean age of the studied groups varied between 52.0 years for group 2 and 55.30 years for group 4. There was a statistically significant difference among studied groups regarding location (*p* < 0.001); while there was no statistically significant difference regarding age and sex (*p* value: 0.464 and 0.353, respectively).

**Figure 1.**
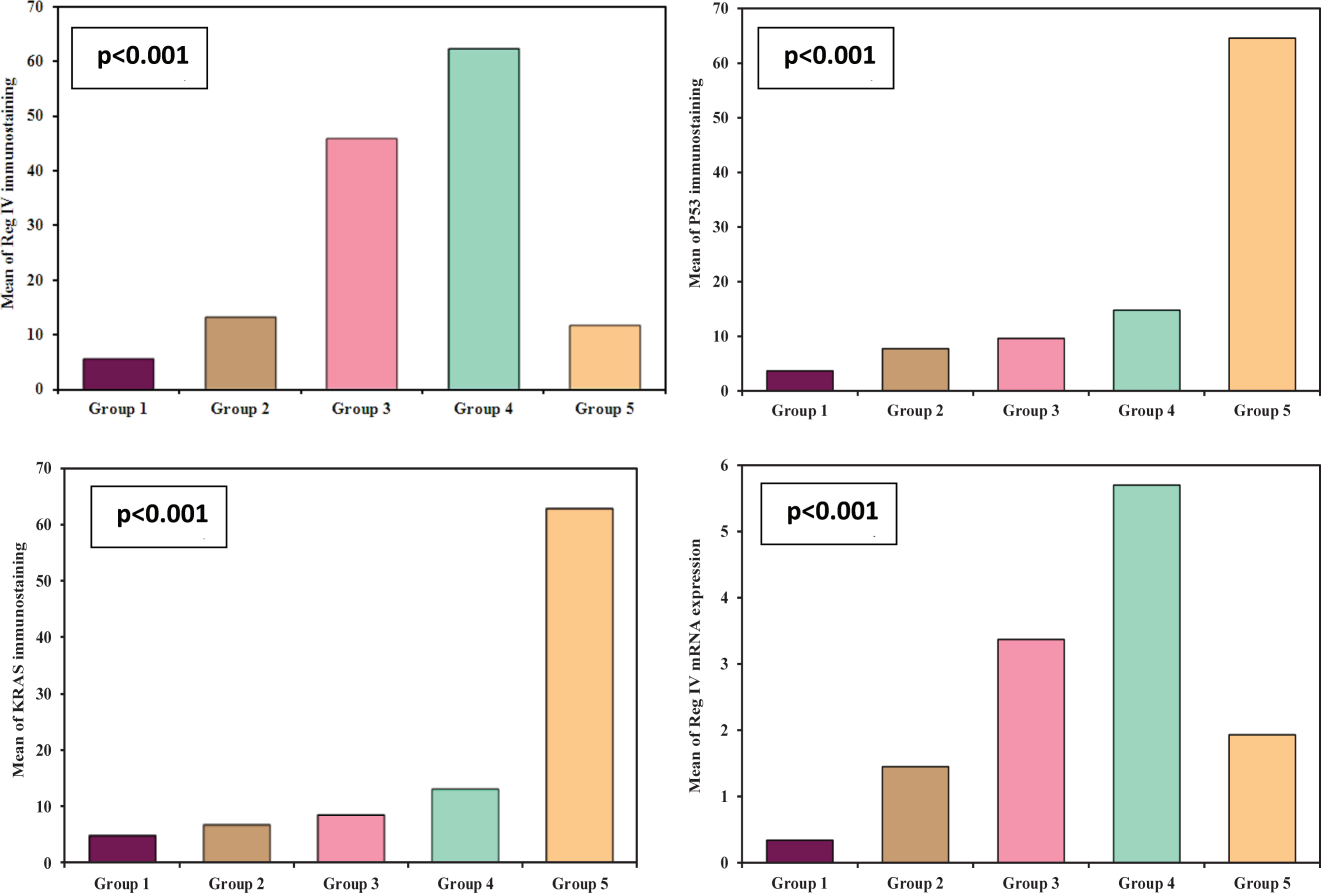
Immunostaining of studied markers and Reg IV mRNA expression in between the five studied groups.

Regarding the molecular analysis of studied cases, Reg IV staining was highly expressed in groups 3 and 4 (mean 45.80 and 62.35, respectively). On the other hand, P53 and KRAS immunostaining were highly expressed in group 5 (mean, 64.57 and 62.90). Reg IV mRNA expression was significantly higher in groups 3 and 4 (mean 3.37 and 5.70, respectively). All these findings were statistically significant between the five studied groups (*p* value < 0.001). These findings were all summarized in Table 2 and Figure 1.

**Figure 2.**
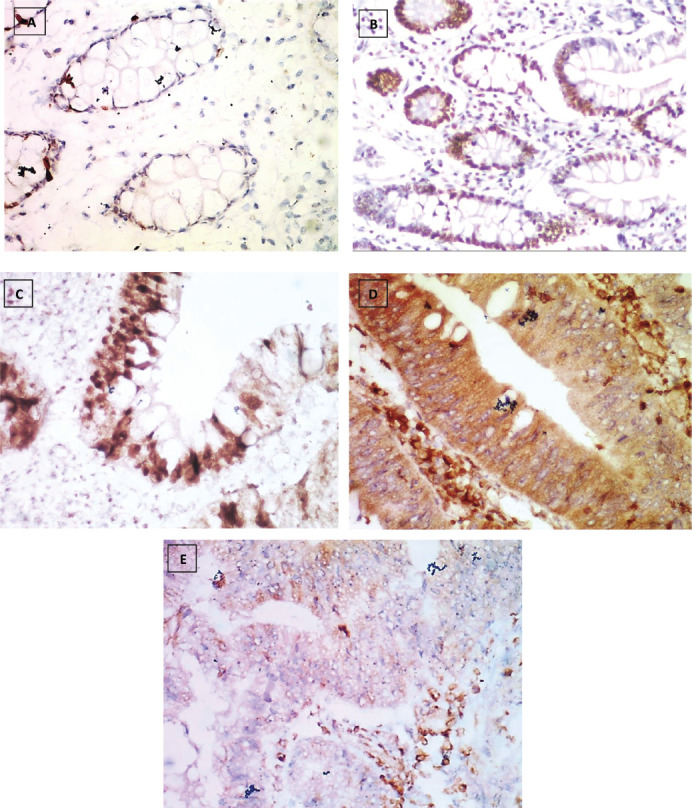
Reg IV immunostaining, (a): Negative expression in normal mucosa (group 1) 0.87% score 0, (b): Positive expression in UC case (group 2) 43.61% score 1, (c): Positive expression in UC-dysplasia case (group 3) 58.46% score 2, (d): Positive expression in UC-CRC case (group 4) 82.23% score and (e): Negative expression in S-CRC case (group 5) 6.15% score 0 (×400).

**Figure 3.**
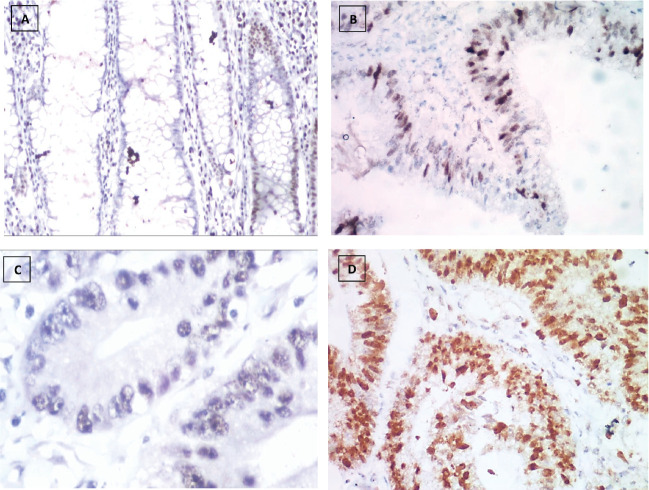
P53 immunostaining, (a): Negative expression in UC case (group 2) 2.45% score 0, (b): Positive expression in UC-dysplasia case (group 3) 26.18% score 1, (c): Negative expression in UC-CRC case (group 4) 3.47% score 0 and (d): Positive expression in S-CRC case (group 5) 76.34% score 2 (×400).

**Figure 4.**
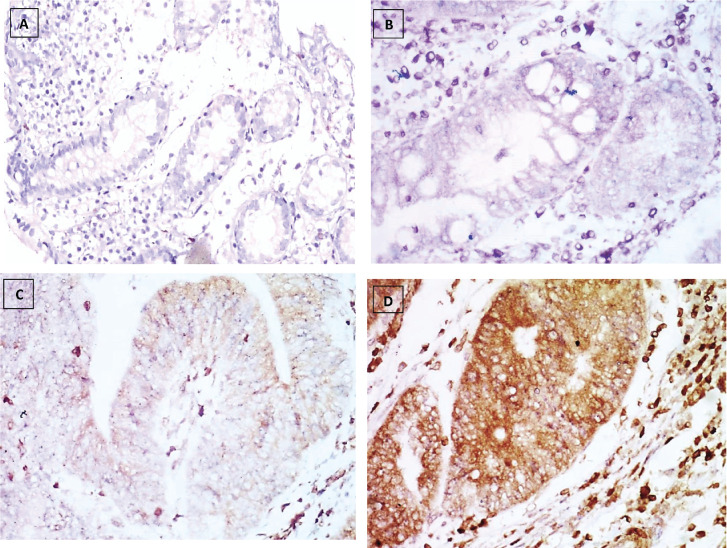
KRAS immunostaining, (a): Negative expression in UC case (group 2) 0.74% score 0, (b): Negative expression in UC-dysplasia case (group 3) 2.81% score 0, (c): Negative expression in UC-CRC case (group 4) 7.34% score 0 and (d): Positive expression in S-CRC case (group 5) 81.04% score 2 (×400).

The number of stained cells was scored as previously described in to three scores (Figures 2–4). Higher staining scores for Reg IV expression were found in groups 3 and 4 while the higher staining scores for P53 and KRAS were found in group 5. Regarding group 1, the staining score of all studied markers was mainly of score 0 (90% for Reg IV and 100% for each P53 and KRAS). For group 2, the staining score of Reg IV was mainly of score 1 (55%) and score 0 for both P53 and KRAS (90% and 95%, respectively). For group 3, the staining score of Reg IV was mainly of score 2 (45%) and score 0 for both P53 and KRAS (85% and 90%, respectively). For group 4, the staining score of Reg IV was mainly of score 3 (85%) and score 0 for both P53 and KRAS (70% and 80%, respectively). For group 5, the staining score of Reg IV was mainly of score 0 (80%) and score 2 for both P53 and KRAS (90% and 85%, respectively).

All these findings were statistically significant between the five studied groups (*p* value < 0.001). These results are summarized in Table 3.

There was a negative correlation between Reg IV immunoexpression and P53 expression in both group 4 and group 5 (−0.537 and −0.106, respectively). Moreover, there was a negative correlation between Reg IV immunoexpression and KRAS in groups 4 and 5 (−0.792 and −0.189, respectively) as shown in Table 4.

**Figure 5.**
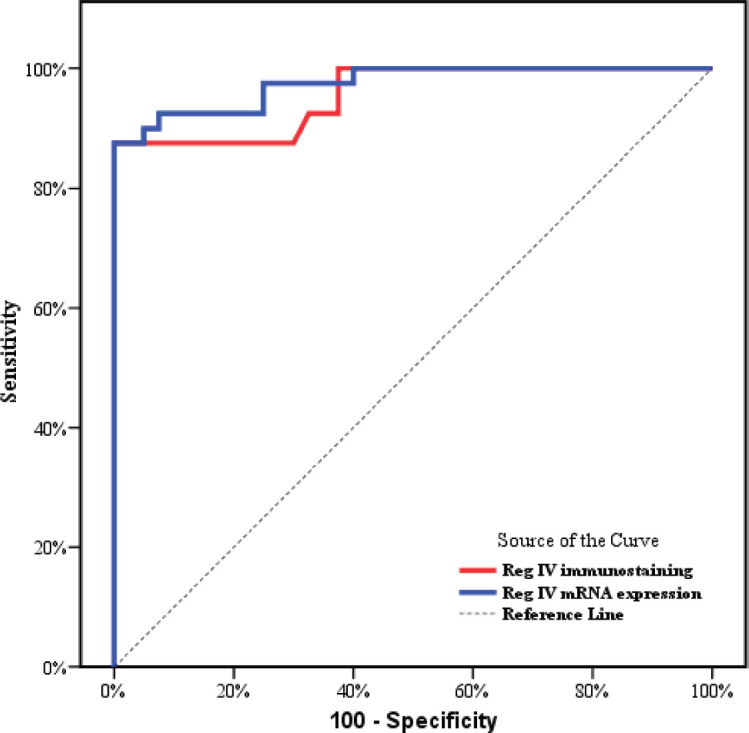
ROC curve for Reg IV immunostaining and mRNA expression to discriminate group 3 + 4 (n = 40) from group 1 + 2 (n = 40).

As described before P53 and KRAS immunostaining was mainly negative (score 0) in groups 3 and 4 so, the ROC curve analysis was carried out to evaluate the validity of the Reg IV in discriminating between neoplastic tissue associated with UC (groups 3 + 4) from non-neoplastic colonic mucosa (groups 1 + 2) as illustrated in Table 5 and Figure 5. The sensitivity of Reg IV immunoexpression and mRNA expression was 87.5% and 92.5%, respectively. The specificity of Reg IV immunoexpression and mRNA expression was 85% and 90%, respectively. The cut off values for Reg IV and Reg IV mRNA expression were more than 17.93% and 2.01%, respectively.

## Discussion

UC-CRC is frequently diagnosed at an advanced stage and due to the higher mortality rate and worse prognosis in UC-CRC compared to S-CRC, early prediction of UC-CRC is crucial [4]. Although traditional endoscopic screening has been conducted for years, its efficiency primarily depends on operator experience and improvements in the endoscopic facility. Hence, molecular monitoring have an enormous potential to enhance the clinical management of the UC-N [3].

In the current study, our results demonstrated that Reg IV mRNA and immunohistochemical expressions in colorectal tissue were significantly elevated in UC-dysplasia and UC-CRC patients in comparison to normal colorectal tissue, UC and S-CRC groups, which may suggest the significance of Reg IV in the development of colitic cancer from UC mucosa. These findings were consistent with Nanakin *et al* [19] who revealed that REG IV mRNA was strongly enhanced in dysplasia and cancerous lesions in UC tissues. Previous studies have also reported the overexpression of Reg IV gene in colorectal adenocarcinoma, gastric cancer and pancreatic cancer [12, 13, 15].

Reg IV may be involved in colitic cancer development through its growth-promoting action. The basic biological functions of Reg family proteins include the induction of cell proliferation, cell migration, cell growth and cell adhesion [20]. Reg IV protein was reported to have antiapoptotic as well as mitogenic effects on colon cancer cells, via activating Akt signaling [19]. Therefore, Reg IV may play a mitogenic and/or antiapoptotic role in CRC development.

*In vitro* studies have revealed that Reg IV expression was induced by hepatocyte growth factor and basic fibroblast growth factor via the MAPK-dependent pathway. Moreover, colon cancer cells overexpressing REG IV gained significant growth ability [21]. Taken together, these findings imply that REG IV may promote epithelial cell proliferation in the UC-colitic cancer sequence.

Reg IV may also promote carcinogenesis by activating the epidermal growth factor receptor (EGFR), as Reg IV is a strong stimulator of the EGFR/Akt/activator protein-1 (AP-1) signaling pathway in colon cancer cells [22]. Therefore, suppressing endogenous Reg IV expression or inhibiting Reg IV/EGFR signaling may have the potential as a novel therapeutic tool to prevent dysplasia and cancer associated with UC.

In this study, P53 and KRAS immunostaining were highly expressed in S-CRC patients compared to UC-CRC. This was in agreement with past studies [23, 24]. Furthermore, Reg IV immunoexpression had shown a negative correlation with P53 and KRAS immunoexpression in UC-CRC and S-CRC groups. As demonstrated in many studies S-CRC develops with a sequence of molecular events, including alterations of KRAS, p53, adenomatous polyposis coli; however, molecular events leading to carcinogenesis in UC-CRC and S-CRC may be different [25, 26].

On the contrary, UC-CRC develops through an inflammation–dysplasia–carcinoma pathway. The longer the duration of UC is, the greater the risk of CRC [27]. This could be explained by the fact that prolonged inflammation can cause immunological dysfunction, the release of inflammatory cytokines and epigenetic alterations, all of which can result in dysplasia and cancer. This highlights the critical role that chronic inflammation plays in the pathogenesis of UC-CRC [28]. 

From the aforementioned results, it can be implied that targeting the KRAS/p53 pathway may not be an effective therapy in UC-CRC as opposed to S-CRC. However, Reg IV targeting could be a potential therapeutic tool in preventing dysplasia and cancer associated with UC.

ROC curve analysis was applied to assess the power Reg IV expression in differentiating UC-dysplasia and colitic cancer from normal colonic mucosa and UC groups. According to the ROC curve in our study, the sensitivity of Reg IV mRNA expression and immunostaining were 92.50% and 87.50%, respectively, and the specificity was 90.0% and 85.0%, respectively. The cut off of Reg IV mRNA relative expression and immunostaining in predicting dysplasia and colitic cancer in UC patients was found to be >2.01 and >17.93, respectively.

Our study revealed that Reg IV was up-regulated in UC-CRC tissues than in adjacent normal mucosa indicating that Reg IV overexpression may be an early event in colorectal carcinogenesis in long-standing UC patients. Most studies [11, 12, 19, 20] as well as the current study estimated Reg IV levels by immunohistochemistry staining and qRT‐PCR in tissue specimens, additionally Oue *et al* [29] used the enzyme-linked immunosorbent assay for Reg IV serum levels. Testing for Reg IV in the blood can potentially detect its level throughout the body; however, it does not indicate its definitive relation with UC or carcinoma in the colonic mucosa. Therefore, Reg IV expression should be investigated in colonic mucosa tissue samples for proper diagnosis [12, 19].

In conclusion, our results suggest that UC-dysplasia, UC-CRC and S-CRC may have different molecular pathways given the higher expression of Reg IV but lower KRAS and P53 expression in patients with UC-CRC in comparison to S-CRC. Our findings indicate the promising role of Reg IV as candidate biomarker for dysplasia and cancer screening in patients with UC. Hopefully, understanding the molecular pathway unique to UC-CRC may provide a useful tool for targeted therapy either by reducing endogenous Reg IV expression or blocking Reg IV downstream signaling. Ultimately, further clinical larger scale studies are needed to validate our findings.

## Limitation of the study

There are some limitations in our study; the sample size is relatively small; therefore, future studies with bigger sample sizes are recommended to corroborate the current experimental results and to validate more anti-cancer agents that could target the Reg IV/EGFR/Akt/AP-1 signaling pathway.

## Conflicts of interest

There is no conflict of interest.

## Financial support and sponsorship

Nil.

## Figures and Tables

**Table 1.** Clinico-pathological characteristics in the five studied groups.

	Group 1 (*n* = 20)	Group 2 (*n* = 20)	Group 3 (*n* = 20)	Group 4 (*n* = 20)	Group 5 (*n* = 20)	Test of Sig.	*p*
**Sex**							
** Male**	10 (50%)	14 (70%)	14 (70%)	16 (80%)	14 (70%)	*χ*^2^ = 4.412	0.353
** Female**	10 (50%)	6 (30%)	6 (30%)	4 (20%)	6 (30%)		
**Location**							
** Left**	7 (35%)	7 (35%)	7 (35%)	5 (25%)	10 (50%)	*χ*^2^ = 33.519^*^	^MC^*p* < 0.001^*^
** Right**	9 (45%)	2 (10%)	0 (0%)	9 (45%)	7 (35%)		
** Pancolitis**	0 (0%)	3 (15%)	7 (35%)	0 (0%)	0 (0%)		
** Rectum**	4 (20%)	8 (40%)	6 (30%)	6 (30%)	3 (15%)		
**Age**							
** Mean ± SD.**	52.55 ± 5.99	52.0 ± 5.28	53.10 ± 6.05	55.30 ± 7.15	54.35 ± 7.0	*F* = 0.906	0.464
** Median (Min. – Max.)**	52.0 (38.0–63.0)	52.50 (39.0–67.0)	53.0 (45.0–67.0)	52.50 (46.0–70.0)	52.50 (44.0–72.0)		

SD: Standard deviation; *F* for One way ANOVA test; *c*^2^: Chi square test; MC: Monte Carlo; p: *p* value for comparing between the different studied groups

Group 1: Normal mucosa; Group 2: UC; Group 3: UC-dysplasia; Group 4: UC-CRC; Group 5: S-CRC

**Table 2.** Immunostaining of studied markers and Reg IV mRNA expression (molecular analysis) in between the five studied groups.

	Group 1 (*n* = 20)	Group 2 (*n* = 20)	Group 3 (*n* = 20)	Group 4 (*n* = 20)	Group 5 (*n* = 20)	Test of Sig.	*p*
**Reg IV immunostaining**							
**Mean ± SD.**	5.67 ± 2.32	13.17 ± 6.16	45.80 ± 18.77	62.35 ± 20.11	11.73 ± 7.91	*F* = 71.858^*^	<0.001^*^
**Median (Min. – Max.)**	5.36 (2.47–10.95)	13.61 (4.98–23.87)	48.05 (8.76–66.98)	65.96 (8.90–85.87)	8.68 (4.90–30.65)		
**p0**		0.378	<0.001^*^	<0.001^*^	0.592		
**p1**			<0.001^*^	<0.001^*^	0.997		
**Sig. bet. grps.**			p_2_ = 0.001^*^,p_3_ < 0.001^*^,p_4_ < 0.001^*^				
**P53 immunostaining**							
**Mean ± SD.**	3.68 ± 1.05	7.70 ± 2.80	9.62 ± 4.08	14.76 ± 10.43	64.57 ± 8.57	*F* = 305.387^*^	<0.001^*^
**Median (Min. – Max.)**	3.68 (1.76–5.56)	7.63 (3.23–15.23)	8.65 (3.98–19.76)	9.82 (7.8–45.87)	65.96 (46.98–76.87)		
**p0**		0.287	0.035^*^	<0.001^*^	<0.001^*^		
**p1**			0.880	0.007^*^	<0.001^*^		
**Sig. bet. grps.**			p_2_ = 0.095,p_3_ < 0.001^*^,p_4_ < 0.001^*^				
**KRAS immunostaining**							
**Mean ± SD**	4.84 ± 1.61	6.73 ± 2.75	8.48 ± 3.04	13.04 ± 10.97	62.90 ± 8.97	*H* = 65.575^*^	<0.001^*^
**Median (Min. – Max.)**	4.76 (2.71–9.65)	6.67 (2.54–12.23)	8.65 (2.76–16.23)	8.76 (5.07–47.87)	62.71 (46.06–77.98)		
**p0**		0.079	0.002^*^	<0.001^*^	<0.001^*^		
**p1**			0.174	0.026^*^	<0.001^*^		
**Sig. bet. grps.**			p_2_ = 0.388,p_3_ < 0.001^*^,p_4_ < 0.001^*^				
**Reg IV mRNA expression**							
**Mean ± SD.**	0.34 ± 0.32	1.45 ± 0.74	3.37 ± 1.16	5.70 ± 1.22	1.93 ± 0.82	*H* = 79.315^*^	<0.001^*^
**Median (Min. – Max.)**	0.21 (0.03–0.87)	1.53 (0.09–2.47)	3.91 (0.91–4.91)	5.80 (2.43–7.76)	1.95 (0.32–3.12)		
**p0**		0.006^*^	<0.001^*^	<0.001^*^	<0.001^*^		
**p1**			0.002^*^	<0.001^*^	0.325		
**Sig. bet. grps.**			p_2_ = 0.017^*^,p_3_ = 0.030^*^,p_4_ < 0.001^*^				

SD: Standard deviation; *F* for One way ANOVA test; *H* for Kruskal Wallis test

p: *p* value for comparing between the different studied groups

p_0_: for comparing between Group 1 and each other group

p_1_: for comparing between Group 2 and each other group

p_2_: for comparing between Group 3 and Group 4

p_3_: for comparing between Group 3 and Group 5

p_4_: comparing between Group 4 and Group 5

Group 1: Normal mucosa; Group 2: UC; Group 3: UC-dysplasia; Group 4: UC-CRC; Group 5: S-CRC

**Table 3.** The score of Reg IV, P53 and KRAS immunostaining among the five studied groups.

	Group 1 (*n* = 20)	Group 2 (*n* = 20)	Group 3 (*n* = 20)	Group 4 (*n* = 20)	Group 5 (*n* = 20)	Test of Sig.	*p*
**Reg IV immunostaining**							
**Reg IV score**							
** 0**	18 (90%)	9 (45%)	3 (15%)	2 (10%)	16 (80%)	*χ*^2^ = 80.978^*^	<0.001^*^
** 1**	2 (10%)	11 (55%)	8 (40%)	1 (5%)	4 (20%)		
** 2**	0 (0%)	0 (0%)	9 (45%)	17 (85%)	0 (0%)		
**P53 immunostaining**							
**P53 score**							
** 0**	20 (100%)	18 (90%)	17 (85%)	14 (70%)	0 (0%)	*χ*^2^ = 82.061^*^	^MC^*p* <0.001^*^
** 1**	0 (0%)	2 (10%)	3 (15%)	6 (30%)	2 (10%)		
** 2**	0 (0%)	0 (0%)	0 (0%)	0 (0%)	18 (90%)		
**KRAS immunostaining**							
**KRAS score**						*χ*^2^ = 78.295^*^	^MC^*p* <0.001^*^
** 0**	20 (100%)	19 (95%)	18 (90%)	16 (80%)	0 (0%)		
** 1**	0 (0%)	1 (5%)	2 (10%)	4 (20%)	3 (15%)		
** 2**	0 (0%)	0 (0%)	0 (0%)	0 (0%)	17 (85%)		

*c*^2^: Chi square test; MC: Monte Carlo

Group 1: Normal mucosa; Group 2: UC; Group 3: UC-dysplasia; Group 4: UC-CRC; Group 5: S-CRC

**Table 4.** Correlation between Reg IV versus P53 and KRAS in each group.

Immunostaining	Group 1		Group 2		Group 3		Group 4		Group 5	
	*r*	*p*	*r*	*p*	*r*	*p*	*r*	*p*	*r*	*p*
Reg IV versus P53	0.331	0.153	0.279	0.233	0.183	0.440	−0.537	0.015*	−0.106	0.657
Reg IV versus KRAS	0.100	0.676	0.598*	0.005*	0.286	0.221	−0.792	<0.001*	−0.189	0.426

* Statistically significant at *p* ≤ 0.05

^r^Pearson coefficient

**Table 5.** Diagnostic performance for Reg IV to discriminate between group 3 + 4 (*n* = 40) from group 1 + 2 (*n* = 40).

	AUC	*p*	95% C.I	Cut off	Sensitivity	Specificity	PPV	NPV
Reg IV immunostaining	0.956	<0.001^*^	0.915–0.997	>17.93	87.50	85.0	85.4	87.2
Reg IV mRNA expression	0.974	<0.001^*^	0.946–1.0	>2.01	92.50	90.0	90.2	92.3

* Statistically significant at *p* ≤ 0.05

AUC: Area under a curve; *p* value: Probability value; CI: Confidence Intervals; NPV: Negative predictive value; PPV: Positive predictive value
